# Activated Alk triggers prolonged neurogenesis and Ret upregulation providing a therapeutic target in ALK-mutated neuroblastoma

**DOI:** 10.18632/oncotarget.1883

**Published:** 2014-04-02

**Authors:** Alex Cazes, Lucille Lopez-Delisle, Konstantina Tsarovina, Cécile Pierre-Eugène, Katleen De Preter, Michel Peuchmaur, André Nicolas, Claire Provost, Caroline Louis-Brennetot, Romain Daveau, Candy Kumps, Ilaria Cascone, Gudrun Schleiermacher, Aurélie Prignon, Frank Speleman, Hermann Rohrer, Olivier Delattre, Isabelle Janoueix-Lerosey

**Affiliations:** ^1^ Inserm U830, 26 rue d'Ulm, 75005 Paris, France; ^2^ Institut Curie, Centre de Recherche, 26 rue d'Ulm, 75005 Paris, France; ^3^ Research Group Developmental Neurobiology, Max Planck Institute for Brain Research, Max-von-Laue-Str. 4, 60438 Frankfurt/M, Germany; ^4^ Center for Medical Genetics, Ghent University Hospital, De Pintelaan 185, B-9000 Ghent, Belgium; ^5^ Departement of Pathology, AP-HP, Hôpital Universitaire Robert Debré, 48 boulevard Sérurier, 75019 Paris, France; ^6^ Université Diderot Paris 7, Paris Sorbonne Cité, Paris, France; ^7^ Platform of Experimental Pathology, Institut Curie, 26 rue d'Ulm, 75005 Paris, France; ^8^ LIMP (Laboratoire d'Imagerie Moléculaire Positonique), Hôpital Tenon, 4 rue de la Chine, 75020 Paris, France; ^9^ Laboratoire CRRET, EAC CNRS 7149, Université Paris 12-Val de Marne, 61, avenue du Général de Gaulle, 94010 Créteil, France; ^10^ Institut Curie, Département de Pédiatrie, 26 rue d'Ulm, 75005 Paris, France

**Keywords:** Neuroblastoma, ALK, neurogenesis, therapeutic target, RET

## Abstract

Activating mutations of the *ALK* (Anaplastic lymphoma Kinase) gene have been identified in sporadic and familial cases of neuroblastoma, a cancer of early childhood arising from the sympathetic nervous system (SNS). To decipher ALK function in neuroblastoma predisposition and oncogenesis, we have characterized knock-in (KI) mice bearing the two most frequent mutations observed in neuroblastoma patients. A dramatic enlargement of sympathetic ganglia is observed in *Alk^F1178L^* mice from embryonic to adult stages associated with an increased proliferation of sympathetic neuroblasts from E14.5 to birth. In a *MYCN* transgenic context, the F1178L mutation displays a higher oncogenic potential than the R1279Q mutation as evident from a shorter latency of tumor onset. We show that tumors expressing the R1279Q mutation are sensitive to ALK inhibition upon crizotinib treatment. Furthermore, our data provide evidence that activated ALK triggers *RET* upregulation in mouse sympathetic ganglia at birth as well as in murine and human neuroblastoma. Using vandetanib, we show that RET inhibition strongly impairs tumor growth *in vivo* in both *MYCN*/KI *Alk^R1279Q^* and *MYCN*/KI *Alk^F1178L^* mice. Altogether, our findings demonstrate the critical role of activated ALK in SNS development and pathogenesis and identify RET as a therapeutic target in ALK mutated neuroblastoma.

## INTRODUCTION

Neuroblastoma (NB) is an embryonal cancer of the peripheral sympathetic nervous system (SNS) observed in early childhood. It is characterized by a broad spectrum of clinical behaviors [1-3] and is classified into localized or metastatic disease [4,5]. Two main *bona fide* cancer genes, *MYCN* and *ALK*, have been identified as major actors of NB pathogenesis. Amplification of the *MYCN* oncogene is observed in 25% of NB cases and is associated with a poor prognosis [1,6]. Overexpression of *MYCN* in neuroectodermal cells under the tyrosine hydroxylase (TH) promoter leads to NB in mice, demonstrating that MYCN can contribute to neuroblast transformation *in vivo* [7,8]. Whereas the *MYCN* oncogene is involved in NB oncogenesis only at the somatic level, both somatic and germline activating mutations of the *ALK* gene have been identified in sporadic and familial cases, respectively [9-12]. The *ALK* gene encodes a receptor tyrosine kinase preferentially expressed in the developing peripheral and central nervous systems [13-16]. The occurrence of *ALK* mutations in sporadic cases is around 7% with two hotspots at positions R1275 and F1174. A preferential association of F1174L mutants with *MYCN* amplification has been reported in a large meta-analysis [17]. Analysis of NB families revealed that the R1275Q was the most frequent germline mutation whereas no germline mutation affecting the F1174 residue has been reported in such families [10,11,18].

*In vitro*, sympathetic neuroblast proliferation has been shown to be increased upon overexpression of wild-type (Wt) ALK as well as R1275Q and F1174L mutant forms in a culture model of chick embryonic neuroblasts [19]. Moreover, it has been recently reported that F1174L ALK may transform murine neural crest progenitor cells [20]. *In vivo*, oncogenic cooperation between MYCN and ALK^F1174L^ has been documented in a zebrafish model [21] and in transgenic mice [22,23].

Here we report the characterization of the first two lines of knock-in (KI) mice carrying the two most frequent *Alk* activating mutations observed in NB patients. These mice enable to investigate the role of *Alk* mutations in a physiological context, in both development and oncogenesis.

## RESULTS

### Generation of *Alk^R1279Q^* and *Alk^F1178L^* KI mouse lines

**Figure 1:**
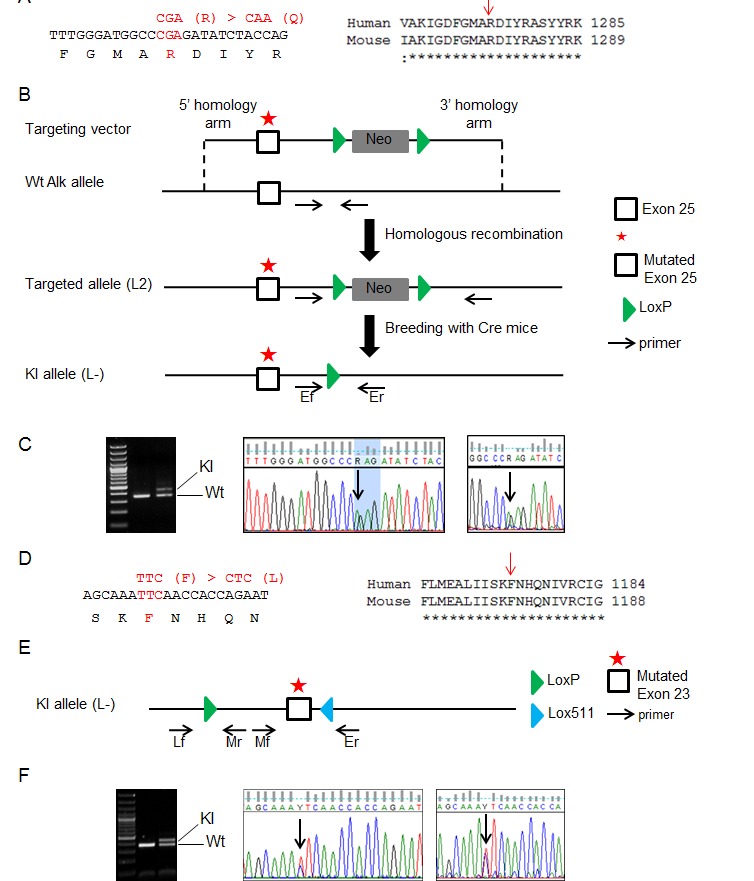
Generation of *Alk^R1279Q^* and *Alk^F1178L^* KI mice (A) Nucleotide and protein sequences surrounding the mutated residue in exon 25 of mouse *Alk*. The R1275 residue in the human ALK receptor corresponds to the R1279 position in the mouse Alk receptor. (B) Schematic representation of the strategy used to obtain*Alk^R1279Q^* KI mice. The Neo cassette was removed *in vivo* by Cre recombination. (C) PCR analysis of genomic tail biopsy DNA using primers Ef and Er detects successful recombination events in the KI mice (left panel). Direct Sanger sequencing on genomic DNA (middle panel) confirmed that one mutated allele was present in heterozygous mice. Heterozygosity resulted in equal amounts of Wt and mutated mRNAs (right panel). (D) Nucleotide and protein sequences surrounding the mutated residue in exon 23 of mouse *Alk*. The F1174 residue in the human ALK receptor corresponds to the F1178 position in the mouse Alk receptor. (E) Schematic representation of the KI allele of the*Alk^F1178L^* KI mice line. (F) The same panels as in (C) are shown for the F1178L mutation. PCR analysis of genomic tail biopsy DNA was performed using primers Lf and Mr to detect the KI allele (left panel).

In order to get insights into the role of the ALK R1275Q and F1174L mutations observed in NB patients, we developed KI mice targeting the corresponding residues in the mouse Alk receptor, *i.e.* R1279Q and F1178L, respectively (Figure 1A,D). For the R1279Q mutation, a targeting vector was constructed as shown in Figure 1B. Homologously recombined ES129 clones were selected and injected into blastocysts. Resulting chimeric mice were crossed with transgenic Cre mice in order to remove the Neo cassette. The Cre transgene was then further segregated yielding one *Alk^R1279Q^* KI mice line. The presence of the mutation was confirmed by direct Sanger sequencing and analysis of SNS ganglia cDNA showed that heterozygosity resulted in balanced amounts of Wt and mutated mRNAs (Figure 1C).

For the F1178L mutation (Figure 1D), we used a different approach (see Methods) that led to a KI allele (L-) bearing the mutated exon 23 flanked by one LoxP and one Lox511 sites (Figure 1E). We confirmed that both the Wt and mutated mRNAs were expressed in *Alk^F1178L^* heterozygous mice (Figure 1F).

### Major size and proliferation abnormalities of sympathetic ganglia in KI *Alk* mice

**Figure 2:**
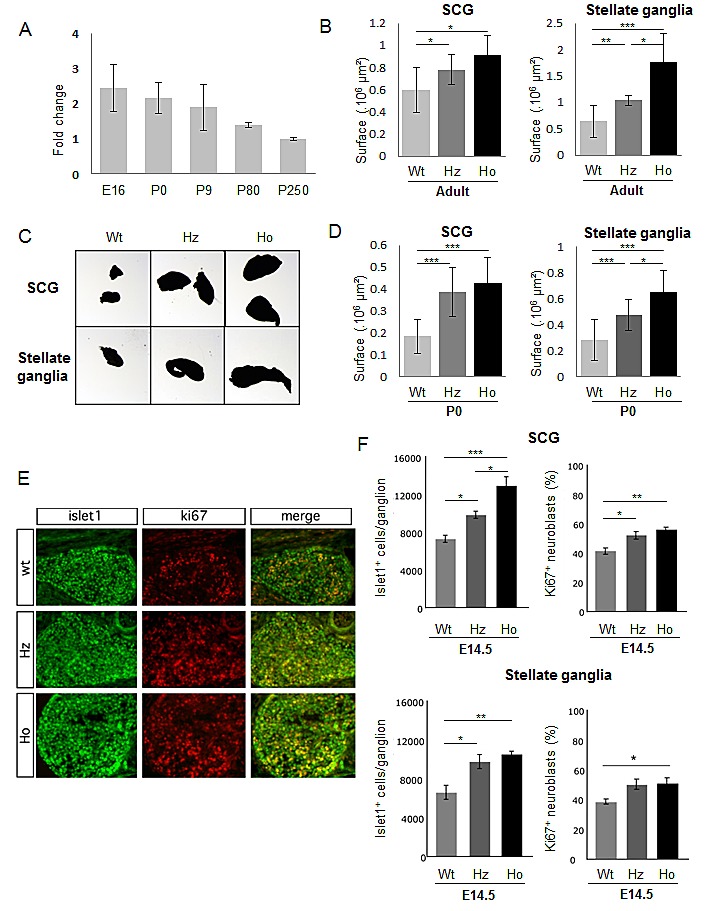
Abnormalities of superior cervical and stellate ganglia in *Alk^F1178L^* KI mice (A) Expression of Alk mRNA in the sympathetic ganglia of Wt animals determined by RT-qPCR from E16 to adult stages. Alk expression was calculated relative to the mean expression at P250. (B) Enlargment of SCG and stellate ganglia in adult *Alk^F1178L^* KI mice. (C) Example of increased size of SCG and stellate ganglia at birth in *Alk^F1178L^* KI mice compared with littermate controls. (D) Quantification of SCG and stellate ganglia size at P0. (E) Immunofluorescence with islet1 and ki67 antibodies was performed on SCG sections of Wt, heterozygous (Hz) and homozygous (Ho) *Alk^F1178L^* embryos at E14.5. (F) Quantification of islet1-positive cells and double positive cells for islet1 and ki67 revealed a significant increase in the number of neuroblasts of the SCG and stellate ganglia in mutant embryos as well as an increased proliferation. Bonferroni Multiple Comparisons Tests were used to evaluate differences between the groups (n=5 samples in each group). Error bars represent standard deviation.

**Figure 3:**
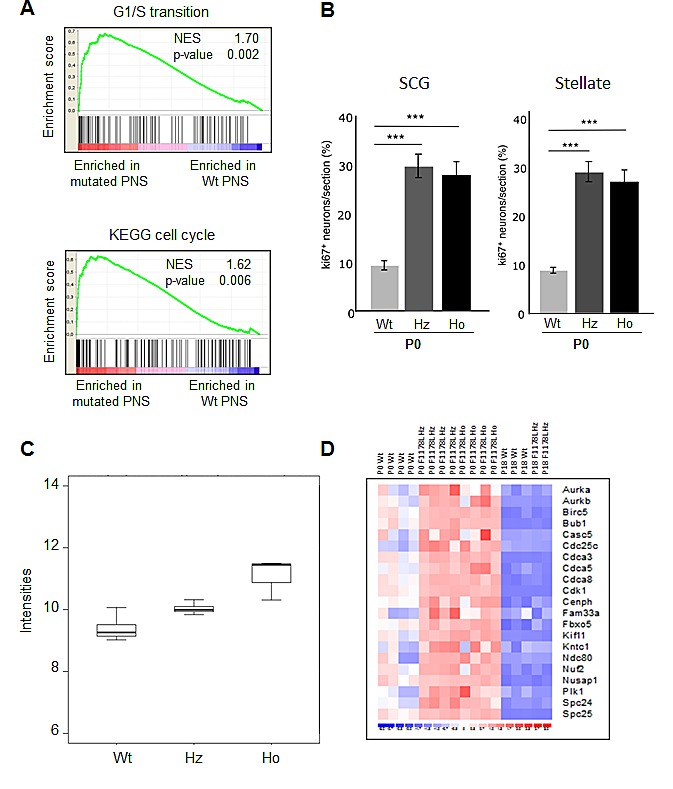
Increased proliferation of sympathetic neurons at P0 in *Alk^F1178L^* KI mice (A) GSEA of cell cycle and G1/S transition pathway genes in transcriptomic data of Wt and mutated ganglia at P0. The normalized enrichment score (NES) and the nominal p-value are indicated. (B) Quantification of double positive cells for islet1 and ki67 revealed an increased proliferation at P0 in ganglia of heterozygotes (Hz) and homozygotes (Ho) KI *Alk^F1178L^* mice. Bonferroni Multiple Comparisons Tests were used to evaluate differences between the groups (n=4 samples in each group). Error bars represent standard deviation. (C) Expression of the *Ret* gene in sympathetic ganglia at birth. (D) Heatmap of genes belonging to the “Mitosis” category (GO:0007067) in P0 and P18 mutated and Wt ganglia.

We first refined *Alk* expression in the SNS by RT-qPCR on mRNAs extracted from superior cervical ganglia (SCG) and stellate ganglia. As shown in Figure 2A, *Alk* expression was highest at E16, and then decreased but remained at adult stage. We then sought to determine whether KI *Alk* mice presented with abnormalities of the sympathetic ganglia. At dissection, an enlargement of the SCG and stellate ganglia was apparent in both *Alk^R1279Q^* and *Alk^F1178L^* mutants. Since this difference was more pronounced in KI *Alk^F1178L^* animals, we subsequently focused on this mutation. We recorded an increased size of the SCG and stellate ganglia in both heterozygotes and homozygotes at the adult stage (Figure 2B) and at birth (Figure 2C,D). This increase was higher in homozygotes than heterozygotes, therefore suggesting a gene dosage effect. At E12.5, we documented a significant increase in the number of neuroblasts (islet1-positive cells) in the SCG and stellate ganglia of homozygous mice compared to Wt (Supplemental Figure 1). In both cases the vast majority of neuroblasts were ki67 positive. Further in development, *i.e.* at E14.5, SCG and stellate ganglia of both heterozygous and homozygous mutant mice presented with a higher number of neuroblasts per ganglion than Wt littermate controls (Figure 2E,F). Interestingly, at that stage, we could document an increased proportion of ki67 positive neuroblasts in Alk mutated ganglia indicating an increased proliferation (Figure 2F). We then performed a transcriptomic profiling of sympathetic ganglia from Wt and KI *Alk^F1178L^* mice, at P0 and P18 stages. At P0, GSEA analysis of Wt versus mutant ganglia revealed a strong signature for cell cycle, S-phase and G1/S transition genes (Figure 3A). Accordingly, ki67 staining showed an excess of proliferation in sympathetic ganglia of KI *Alk^F1178L^* mice (Figure 3B). Recent *in vivo* data suggested a role of Ret in the control of proliferation at late stage neurogenesis [24]. Interestingly, Figure 3C shows that *Ret* expression is increased at P0 in sympathetic ganglia from mutants compared to controls and that this increase is higher in homozygotes compared to heterozygotes (p<0.001, ANOVA test). We observed that the proliferation signature was no more present at P18 (Figure 3D). An enrichment of cell cycle genes was also observed at P0 in sympathetic ganglia of KI *Alk^R1279Q^* mice (Supplemental Figure 2A). As shown in Supplemental Figure 2A, at birth Alk expression was slightly increased in mutated ganglia compared to Wt ganglia, and this increase was somewhat higher in the case of the Alk F1178L mutation compared with the Alk R1279Q mutation. This was confirmed by RT-q-PCR and an increase was also documented at the adult stage in mutants (data not shown). Altogether, our results therefore demonstrate that expression of Alk mutants triggers prolonged neurogenesis in sympathetic ganglia. However, we did not observe any tumor in these KI mice, indicating that activated Alk is not by itself sufficient to induce neuroblastic tumors.

### Both *Alk* R1279Q and F1178L mutations cooperate with *MYCN* to induce NB *in vivo*

**Figure 4:**
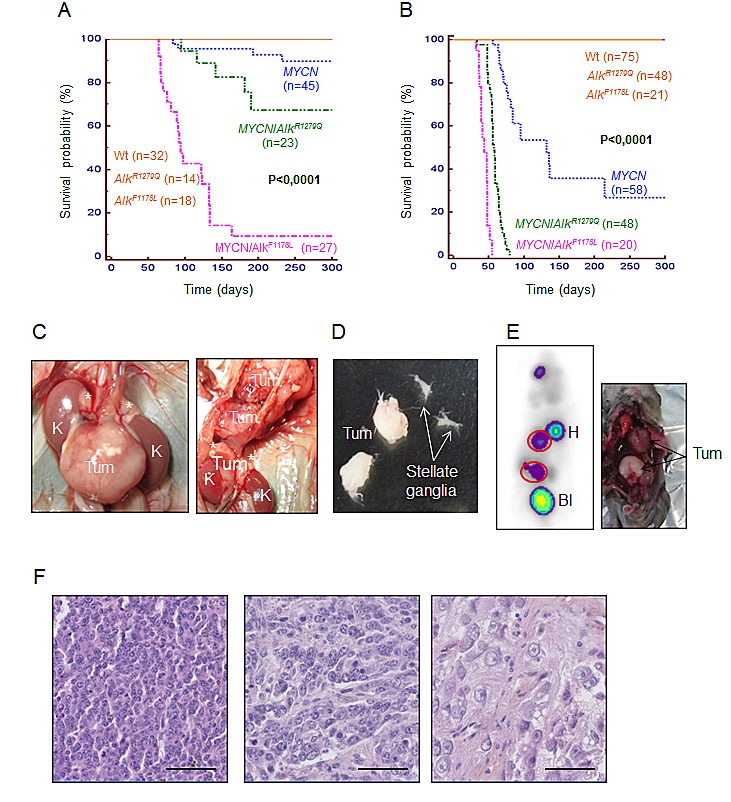
Both *Alk^R1279Q^* and *Alk^F1178L^* mutations cooperate with MYCN *in vivo* to induce NB tumors (A and B) Kaplan-Meier analysis showing that the *Alk^R1279Q^* and *Alk^F1178L^* mutations cooperate with *MYCN* to induce NB in a 129B6F1 (A) and 129S2 (B) background, respectively. 129B6F1: F1 background from 129×1/SvJ × C57Bl6/N intercrosses. Mice were sacrificed upon detection of abdominal palpable tumors or paralysis of the lower extremities. (C) Examples of abdominal and thoracic tumors at autopsy. Tum: tumor; K: kidney. Stars mark the adrenal glands. (D) Example of cervical tumors deriving from the SCG. The stellate ganglia of the same mice are shown next to the cervical tumors. (E) Detection of one multifocal (abdominal and thoracic) tumor in a *MYCN*/KI *Alk^R1279Q^* mice by molecular imaging using ^18^F[FDG] micro-PET. H: heart; Bl: bladder (left panel). Mouse autopsy confirmed the presence of both tumors (right panel). (F) Representative images of tumor histology. Left panel: *MYCN* tumor, undifferentiated; middle and right panels: *MYCN*/*Alk* tumors exhibiting signs of differentiation. Scale bars, 50 μm.

We then explored the oncogenic potential of both mutations in cooperation with the *MYCN* gene by crossing *Alk^R1279Q^* and *Alk^F1178L^* KI mice with *TH-MYCN* mice. Two sets of breedings were analyzed, the first one on a mixed background (F1 between 129×1/SvJ *TH-MYCN* and C57Bl/6 KI *Alk^R1279Q^* or *Alk^F1178L^*) and the second one on a genetic background >93.5 % 129S2. On both backgrounds *Alk^R1279Q^* and *Alk^F1178L^* strongly synergized with *MYCN* overexpression to induce tumors (Figure 4A,B). Full penetrance and much shorter latency were observed in the 129S2 background. Interestingly, tumor onset was even earlier in the case of the F1178L mutation compared with the R1279Q mutation, demonstrating that the oncogenic potential of the F1178L mutation is higher than that of the R1279Q mutation.

In most cases, tumors were detectable through abdominal palpation of tough masses (Figure 4C). Autopsy revealed that abdominal tumors were median, perivascular and locally invasive without evidence of macroscopic tumor spread to other distant organs. Whereas multiple primary tumors were rarely observed in *TH-MYCN* mice, tumors at two or three locations (abdominal/thoracic/cervical) were detected in 70% and 100% of *TH*-*MYCN*/KI *Alk^R1279Q^* and *TH*-*MYCN*/KI *Alk^F1178L^* mice, respectively (Figure 4C,D). Such multifocal tumors could be detected by micro-PET analysis (Figure 4E). Histological analysis using the criteria defined to describe human NB [30] allowed us classifying most *MYCN* tumors as NB, stroma-poor, undifferentiated and *MYCN*/*Alk* tumors as NB, stroma-poor, poorly differentiated or differentiating (Figure 4F). Immunohistochemistry confirmed that all tumors expressed the adrenergic marker TH (Supplemental Figure 3). The vast majority of both types of tumors presented with a high *mitosis*-karyorrhexis index and consistently ki67 staining revealed a high proportion of proliferating cells (Supplemental Figure 3). As expected, Alk and MYCN proteins were expressed in *MYCN/Alk* tumors (Supplemental Figure 4).

A subset of tumors was further characterized by array-CGH. This analysis indicated that genomic alterations, occurring as whole or partial chromosome gains and losses were frequent (7 out of 9) in tumors obtained in *TH-MYCN*/KI *Alk* mice in the mixed background (129×1/SvJ × C57Bl/6) (Table 1). In contrast, no alteration was detected in 9 tumors observed in *TH-MYCN*/KI *Alk* mice in the 129S2 background. These data therefore indicate that the endogenous expression of activated Alk combined with *MYCN* overexpression is sufficient to drive NB tumorigenesis in a genetic background allowing development of neuroblastic tumors. This is consistent with observations reported in double trangenic MYCN/ALK^F1174L^ mice [23].

**Table 1:** Genomic aberrations in *MYCN/Alk* tumors defined by array-CGH. Abnormalities are indicated for each tumor together with the genotype, genetic background, sex and age of the mouse. 129B6F1: F1 background from 129×1/SvJ × C57Bl6/N intercrosses

Tumor	Genotype	Genetic background	Sex	Age in days	Abnormalities in CGH
tumor 31	MYCN / KI AlkF1178L	129S2	F	36	0
tumor 28	MYCN / KI AlkF1178L	129S2	M	37	0
tumor 29	MYCN / KI AlkF1178L	129S2	F	44	0
tumor 30	MYCN / KI AlkF1178L	129S2	M	36	0
tumor 13	MYCN / KI AlkR1279Q	129S2	M	67	0
tumor 14	MYCN / KI AlkR1279Q	129S2	M	75	0
tumor 16	MYCN / KI AlkR1279Q	129S2	M	59	0
tumor 18	MYCN / KI AlkR1279Q	129S2	M	58	0
tumor 19	MYCN / KI AlkR1279Q	129S2	M	59	0
tumor 2	MYCN / KI AlkF1178L	129B6F1	M	133	2- 4- 3+ 6+ 12+ 13- 14- 16-
tumor 1	MYCN / KI AlkF1178L	129B6F1	M	133	11+
tumor 4	MYCN / KI AlkF1178L	129B6F1	F	91	3+ 17+
tumor 11	MYCN / KI AlkF1178L	129B6F1	F	98	0
tumor 38	MYCN / KI AlkF1178L	129B6F1	F	134	3+5-15q-16-
tumor 37	MYCN / KI AlkF1178L	129B6F1	M	64	0
tumor 8	MYCN / KI AlkR1279Q	129B6F1	F	181	4-5-6+10+12+13-14-17+19-
tumor 5	MYCN / KI AlkR1279Q	129B6F1	F	116	4- 4qA3+15qE2-
tumor 9	MYCN / KI AlkR1279Q	129B6F1	M	190	2q+11q+ 15qF3-

### *MYCN*/*Alk* mouse NB express both adrenergic and cholinergic markers

**Figure 5:**
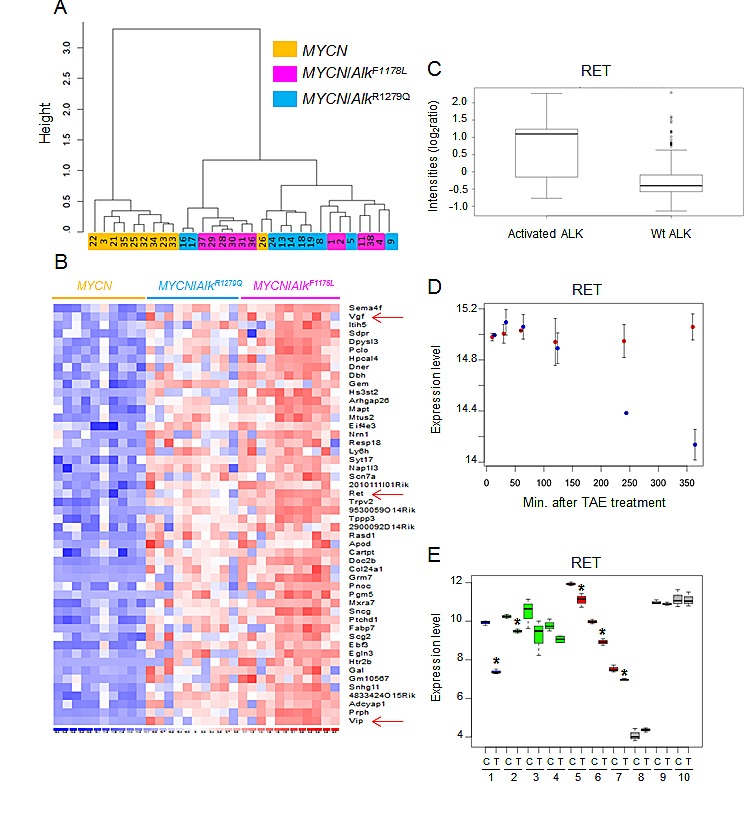
*Ret* is upregulated in murine and human NB exhibiting Alk activation (A) Unsupervised hierarchical clustering using the top 5% genes with the highest IQR clearly separates *MYCN* and *MYCN*/*Alk* tumors. (B) The list of the top 50 upregulated genes in *MYCN*/*Alk* tumors is shown together with a heatmap. It includes the *Ret, Vgf* and *Vip* genes. (C) Expression level of the *RET* gene in primary tumors with (n=16) and without ALK activation (n=144) as determined by Schulte and colleagues [29]. (D) RET down-regulation in CLB-Ga upon TAE-684 treatment (blue). Untreated cells are shown in red. (E) Decreased expression of *RET* upon TAE-684 treatment in human NB cell lines with amplified ALK (blue), F1174L ALK (green) or R1275Q ALK (red). Cell lines with Wt ALK appear in grey. C: Control cells; T: TAE-684 treated cells. 1: NB-1; 2: Kelly; 3: SK-N-SH; 4: SMS-KNCR; 5: CLB-Ga; 6: LAN5; 7: UKF-NB-3; 8: IMR-32; 9: NGP; 10: SK-N-AS. P values were calculated using one-sided Wilcox-test.

Then, we generated expression profiles of *MYCN* and *MYCN*/*Alk* tumors. In agreement with the differences observed through histological analysis, unsupervised hierarchical clustering clearly separated both types of tumors (Figure 5A), with the exception of one *MYCN* tumor. Figure 5B shows the subset of genes presenting with the highest increased expression in *MYCN/Alk* tumors compared with *MYCN* tumors. Among them, we identified *Ret*, previously shown to be increased at birth in PNS ganglia of Alk mutant animals as well as the *Vgf* (non-acronymic, nerve growth factor inducible gene) and *Vip* (Vasoactive intestinal peptide) genes. In addition to its role in proliferation of late stage neurogenesis, Ret signaling has been shown to control cholinergic properties in immature sympathetic neurons and *Vip* is co-expressed in these cells [26]. Moreover, Vgf expression is a hallmark of Ret-signaling hyperactivity [27,28]. Both *MYCN*/*Alk* and *MYCN* tumors presented with a high expression of the adrenergic markers Th, Dbh and Vmat2 (Supplemental Figure 2B). These data therefore indicate that, unlike *MYCN* tumors which are fully adrenergic, *MYCN/Alk* tumors express both adrenergic and cholinergic markers. Further Gene Ontology analysis documented an enrichment of several categories related to neuronal functions, such as neuronal cell body (z-score: 6.43), axon (z-score: 5.69) and synapse (z-score: 4.69) in *MYCN*/*Alk* tumors. Finally, we observed that expression of the murine *Mycn* gene was significantly higher in *MYCN*/*Alk* tumors compared with *MYCN* tumors, as previously described in transgenic *ALK^F1174L^* mice [22]. Interestingly, it was higher in *Alk* F1178L tumors than *Alk* R1279Q tumors and we made the same observation for the *Alk* gene itself (Supplemental Figure 2B).

### ALK activation controls RET expression in human NB

The observation of a strong upregulation of *Ret* in *MYCN*/*Alk* tumors prompted us to investigate *RET* expression in human tumors presenting with and without ALK activation. We took advantage of the data set of Schulte and colleagues, reporting the expression profiles of such tumors [29]. A differential analysis with multiple testing correction identified the *RET* gene as being upregulated in tumors with activated ALK compared with tumors with Wt ALK (P <0.05). A boxplot of *RET* expression level in these tumors is shown in Figure 5C. To further document the link between ALK activation and *RET* expression, ALK inhibition was first achieved in the *ALK* mutated CLB-Ga human NB cell line using the ALK kinase inhibitor TAE-684 [30]. Figure 5D shows that *RET* was strongly down-regulated upon ALK inhibition. Then, a panel of 10 human NB cell lines was treated for 6 hours with TAE-684. As expected, TAE-684 indeed led to downregulation of phosphoALK (data not shown). Strikingly, we observed a strong down-regulation of *RET* and its target gene *VGF* upon TAE-684 treatment in cell lines presenting with activated ALK (by mutation or amplification) but not in cell lines with Wt ALK (Figure 5E and Supplemental Figure 5). For *VIP*, a decreased expression was observed in 3 out of 7 cell lines with activated ALK after TAE-684 treatment (Supplemental Figure 5). By contrast, the expression level of the adrenergic marker *DBH* was not altered by ALK abrogation (Supplemental Figure 5). Altogether, our observations indicate that *RET* expression is controlled by ALK activation.

### *MYCN/Alk* tumors are sensitive to the Ret inhibitor vandetanib

**Figure 6:**
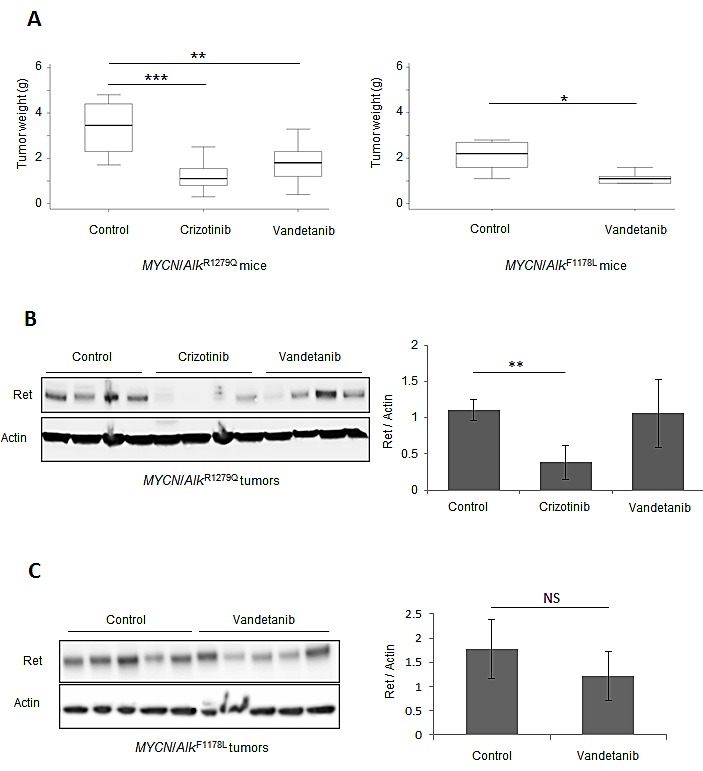
Vandetanib impairs NB growth *in vivo* (A) Left panel, *MYCN*/KI *Alk^R1279Q^* mice were treated for 10 days with control (n=8), crizotinib (n=11) or vandetanib (n=8); right panel, *MYCN*/KI *Alk^F1178L^* mice were treated for 10 days with control (n=5) or vandetanib (n=6). Treatment efficacy was determined by measuring tumor weight at sacrifice. (B) and (C) The Ret expression level was investigated by Western blot in *MYCN*/KI *Alk^R1279Q^* and *MYCN*/KI *Alk^F1178L^* treated tumors and controls using the anti-Ret antibody EPR2871 (Abcam). Actin was used as a standard for quantification.

To further investigate the role of RET in the oncogenic function of activated ALK, we took advantage of vandetanib (ZD-6474), a kinase inhibitor originally described as a second generation EGFR inhibitor but subsequently found to be more potent against VEGFR2 and RET [31]. As shown in Supplemental Figure 6, we documented that Vegfr2 and Egfr were not, or hardly detected in *MYCN*/*Alk* tumors. We first treated *MYCN*/KI *Alk^R1279Q^* mice with palpable abdominal tumors with 100 mg/kg/day crizotinib or 75 mg/kg/day vandetanib by oral gavage. As shown in Figure 6A, after 10 days of treatment we observed a strong reduction in abdominal tumor weight in mice treated either with crizotinib or vandetanib as compared with controls (crizotinib: mean±SD= 1.20±0.18 g *versus* 3.35±0.42 g, p<0.001; vandetanib: mean±SD = 1.79±0.31 g *versus* 3.35±0.42 g, p<0.01). Since the ALK F1174L mutation has been shown to be resistant to crizotinib [22,23,32] we then treated *MYCN*/KI *Alk^F1178L^* mice with vandetanib. We documented a significant decrease in abdominal tumor weight in treated mice (mean±SD = 1.13±0.11 g *versus* 2.08±0.32 g, p<0.05; Figure 6A). These results provide proof of principle that tumors expressing both the *Alk* R1279Q and F1178L mutations are sensitive to Ret inhibition. We further explored the expression level of the Ret protein in non-treated and treated tumors. Figure 6B shows that Ret protein levels are strongly decreased following crizotinib treatment of mice with the R1279Q mutation, which shows that Ret functions as a target of activated Alk *in vivo*. We also evaluated the Ret expression level after vandetanib treatment for both mutations and did not observed any strong difference (Figure 6B,C).

## DISCUSSION

In this study, we investigated the role of physiological level of activated *Alk* in mouse SNS development and NB oncogenesis. In KI *Alk^F1178L^* mice, we characterized an enlargement of the SCG and stellate ganglia from embryonic to adult stages. Further analysis of embryonic and P0 stages indicated that activated Alk affects neurogenesis in sympathetic ganglia in two ways. First, the observation of an increased number of sympathetic neuroblasts in mutant mice at E12.5 suggests that mutated Alk acts at an early stage to elicit either the generation of more neuroblasts from progenitors or an increase of neuroblast proliferation. Second, our data provide evidence that Alk activation induces a prolonged proliferation of sympathetic neurons, already detected at E14.5 and revealed at P0 by transcriptomic analysis and ki67 staining. This abnormal proliferation was no more detectable at P18 and KI mice did not develop NB. This strongly suggests that a second and cooperative genetic event (*e.g. MYCN* amplification) is required to achieve NB tumorigenesis, which is in agreement with the incomplete penetrance of *ALK* mutations in familial cases. Nevertheless, the abnormalities of the sympathetic ganglia of KI *Alk* mice described here may provide a clue for NB predisposition in patients with germline *ALK* mutations.

Recently, an oncogenic cooperation between ALK^F1174L^ and *MYCN* overexpression has been reported in zebrafish and mouse transgenic models [21-23]. Furthermore, *in vitro* studies indicated an higher transforming capacity of ALK F1174L compared with ALK R1275Q [17]. We now show that both F1178L and R1279Q *Alk* mutations accelerate tumor onset and increase tumor penetrance in a *MYCN* context. This is the first demonstration *in vivo* that the R1279Q mutation is able to cooperate with *MYCN* and that the oncogenic potential of the F1178L mutation is higher than that of the R1279Q mutation. This may be related at least in part to the stronger upregulation of murine *Mycn* in *MYCN*/*Alk* tumors bearing the F1178L mutation compared with the R1279Q one, consistently with the reported gene dosage effect of *MYCN* in transgenic mice [8].

Strikingly, we observed that *Ret* expression is controlled by activated Alk not only in *MYCN*/*Alk* mice tumors but also in human NB cell lines presenting with mutation or amplification of the *ALK* gene. In addition, the analysis of the data set of Schulte and colleagues [29] provided strong evidence that RET is also upregulated in human primary tumors exhibiting ALK activation achieved by amplification or point mutation. Interestingly, the *ALK* gene itself is upregulated in human NB presenting with *ALK* mutations [29]. Strikingly, this upregulation is also observed in murine *MYCN*/*Alk* tumors compared with *MYCN* tumors and in the ganglia of mutated animals, in agreement with the reported ALK autoregulation [19,33]. Altogether, these data strongly indicate that tumors obtained in our models are fully relevant to human NB.

Importantly, Ret is known to regulate neuronal migration and axonal growth through the entire SNS [34]. Our observations of an increased *Ret* expression in *MYCN*/*Alk* mice tumors together with an enrichment of genes related to neuronal cell body, axon and synapse categories are therefore in line with the aforementioned roles of Ret in the sympathetic ganglia. Interestingly, abnormalities of the SNS have been reported in mice bearing *Ret* alterations. *Ret^−/-^* mice displayed reduction in the size of the sympathetic ganglia associated with an increased cell death at birth [34], increased cell cycle length at E16.5 and a decreased number of neuroblasts at E18.5 [24]. Conversely in KI *Alk^F1178L^* mice we documented an excess of proliferation in sympathetic ganglia between E14.5 and P0, an increased Ret expression at P0 as well as an enlargement of this tissue. Altogether, these data suggest that activated Alk may exert at least part of its effects on the sympathetic ganglia via Ret signaling.

To further address the role of the RET kinase in the oncogenic function of activated ALK we used vandetanib that has been shown to inhibit RET/MEN2A and RET/MEN2B oncoproteins, as well as RET/PTC3 and Wt RET [35]. We showed that tumors from *MYCN*/KI *Alk^R1279Q^* mice responded to vandetanib *in vivo* with a sensitivity similar to the one measured with the ALK inhibitor crizotinib. In addition, growth of tumors bearing the ALK F1178L mutation, was also impaired by vandetanib. These data therefore indicate that inhibition of only the *Alk* or *Ret* oncogene is sufficient to impair tumor growth, although these tumors also express high level of the *MYCN* gene. Consistently with the *in vitro* data showing that Alk inhibition results in Ret down-regulation in ALK activated NB, we observed a down-regulation of Ret in mice tumors with mutated ALK upon crizotinib treatment.

Crizotinib was recently evaluated in children with refractory solid tumors including NB [36]. However, the limited number of patients with a defined ALK status did not allow drawing strong conclusion regarding crizotinib activity on different ALK mutations. Our demonstration that crizotinib is active *in vivo* in a relevant model of NB corresponding to the R1275Q mutation is of high clinical interest. Interestingly, vandetanib has been shown recently to be well-tolerated and highly active in children with locally advanced or metastatic medullary thyroid cancer in the context of a *RET* M918T mutation [37]. These data may encourage clinicians to consider RET inhibition in NB cases with ALK activation and subsequent high RET expression, particularly in cases presenting with mutations at F1174 exhibiting crizotinib resistance.

## METHODS

### Establishment of the *Alk^R1279Q^* and *Alk^F1178L^* KI mouse lines

*Alk^R1279Q^* and *Alk^F1178L^* KI animals were generated at the Mouse Clinical Institute, Illkirch, France. The R1279 and F1178 positions in the murine Alk receptor correspond to R1275 and F1174 positions in the human ALK receptor, respectively (Figure 1A,D). The targeting vector for *Alk^R1279Q^* (Figure 1B) was constructed as described in Supplementary Methods. Targeted 129Sv/Pas Embryonic Stem (ES) clones were confirmed by PCR and Southern blot and injected into C57BL/6J blastocysts to generate chimeric mice. Chimeras (L2) were crossed with transgenic Cre mice (C57BL/6) to check transmission of the targeted allele in the germline and excise the Neo cassette on F1 progenies (L-). A further cross was performed to segregate the Cre transgene. PCR analysis of genomic tail biopsy DNA was performed as described in Supplementary Methods. For the *Alk^F1178L^* mutation, the detailed procedure is provided in Supplementary Methods.

The care and use of animals used in this study were strictly applying European and National Regulation in force for the Protection of Vertebrate Animals used for Experimental and other Scientific Purposes (Directive 86/609). The protocol complies with internationally established 3R principles, more precisely in accordance with ‘Guidelines for the generation, breeding, care and use of genetically modified and cloned animals for scientific purposes’ (National Health and Medical Research Council NHMRC, Australia, 2007) and the UKCCCR guidelines (Guidelines for the welfare and use of animals in cancer research) [38].

### RT-qPCR

First strand cDNA synthesis was performed on 1 μg of total RNA by use of High capacity cDNA reverse transcription kit (Applied Biosystems). Alk and actin mouse cDNA expression were determined using TaqMan^®^ Universal Master Mix and the TaqMan^®^ gene expression assays Mm01226182-m1 and 4352341E (Applied Biosystems), respectively.

### *TH*-*MYCN* mice

*TH*-*MYCN* mice used in this study have been previously described [8]. Mice on a 129×1/SvJ background were obtained from the NCI mouse repository (http://mouse.ncifcrf.gov/) and further backcrossed on 129S2/SvPasCrl background (abbreviated 129S2, Charles River). Genotyping was performed as previously described [39].

### Tumor histology and immunohistochemistry

Mice tumors were dissected at sacrifice, fixed in a mix of ethanol-acetic acid-formol and paraffin-embedded. Tissue sections were stained with hematoxylin, eosin and safran (HES) and evaluated by a pediatric pathologist. Histochemical analysis was performed with ki67 (SC, #7846; dilution 1/300) and TH (Aves, #TYH; dilution 1/100) antibodies.

### Analysis of sympathetic ganglia

Animals were killed and immediately dissected to collect SCG and stellate ganglia. Ganglia were fixed and surface was evaluated (x2.5, Leica DMR, Vega v2.1 ClaraVision software). Ganglia were then included in agarose and paraffin-embedded. Alternatively, they were fixed for immunostaining or immediately frozen in liquid nitrogen for RNA extraction.

### Immunostaining and quantification of islet1- and ki67-positive cells

E12.5 and E14.5 mouse embryos and P0 stages were fixed in 4% paraformaldehyde overnight, washed with PBS and immersed in 30% sucrose for 24 h. After embedding in Tissue Tek 12 μm cryosections were collected. After antigen retrieval in 10 mM sodium citrate buffer pH 6.0 at 95°C for 20 min sections were incubated overnight at 4°C in PBS/10% FCS/1% BSA with anti-mouse ki67 (BioLegend) and mouse anti-islet1 (Developmental Studies Hybridoma Bank) diluted 1:200 and 1:40, respectively. Alexa 594 anti-rat and Alexa 488 anti-mouse were used as secondary antibodies. To estimate the number of islet1-positive cells, the number of islet1-positive cells in the ganglion area were counted from every sixth section. The density of islet1-positive cells was determined from counts of positive cells. To derive the number of islet1-positive cells /ganglion, counted cells are transformed to counts per volume and corrected according to the Linderstrom-Lang/Abercrombie equation [40].

### ^18^F[FDG] PET Imaging

Micro-PET analysis was performed as described in Supplementary Methods.

### Transcriptomic profiling of mice samples

Transcriptomic profiling of tumors and sympathetic ganglia was performed using Affymetrix Mouse Genome 430 2.0 arrays according to the manufacturer's instructions. RNA was collected from tumors using the miRNeasy kit (Qiagen) and from ganglia (pooled SCG and stellate ganglia) using Trizol^®^ and RNA precipitation. Arrays were normalized by the GCRMA procedure using Brainarray annotations [41].

### Genomic profiling

Genomic DNA from mouse tumors was analyzed versus reference normal DNA. The samples were labeled and co-hybridized to the NimbleGen Mouse CGH 3×720K WG-T arrays according to the manufacturer's protocol. Arrays were washed and then scanned on a GenePix 4000B Scanner using GenePix 5.0 software. Raw data were normalized using NimbleScan v2.5 software (Roche NimbleGen). The normalized data were processed using NimbleScan softwares.

### Treatment of human NB cell lines with TAE-684 and expression analysis

Human NB cell lines were genotyped by DNA fingerprinting (PowerPlex, Promega) and have been previously described [42]. They were grown in RPMI 1640 medium (Invitrogen) supplemented with 10% FCS and antibiotics. They were treated in triplicate with 0.3 μM TAE-684 (Novartis) or DMSO (VWR) for 6 hours. Then expression profiling on the Affymetrix HG-U133PLUS2 platform was performed according to the manufacturer's protocol. The CLB-Ga cell line was treated with 0.3 μM TAE-684 and harvested in duplicate at 6 different time points (10', 30', 60', 120', 240' and 360') for subsequent expression profiling on a custom Agilent 44k expression array using labeling and hybridization protocols of the manufacturer. Affymetrix array data were normalized using the RMA method, while Agilent expression profiling data were normalized using VSN (variance stabilization and normalization) in R packages affy and limma, respectively.

### *In vivo* crizotinib and vandetanib treatment

Crizotinib and vandetanib were purchased from Medchem express. *TH-MYCN*/KI *Alk^R1279Q^* mice with palpable tumors (49 days old) were treated with 100 mg/kg/day crizotinib or 75 mg/kg/day vandetanib by oral gavage. *TH-MYCN*/KI *Alk^F1178L^* mice with palpable tumors (36 days old) were treated with 75 mg/kg/day vandetanib.

### Statistical analysis

Unless otherwise specified, two-tailed Student's t test was used to assay significance. Single, double and triple asteriks indicate statistically significant differences: * P ≤ 0.05; ** P ≤ 0.01; ***, P ≤ 0.001. Survival curves were constructed according to the Kaplan-Meier method and compared using the log-rank test.

### Data deposition

The GEO public database accession numbers for the microarrays data are GSE46583 and GSE46584 for tumors and sympathetic ganglia, respectively.

## SUPPLEMENTARY METHODS AND FIGURES




