# Supplementary material for: Activated Alk triggers prolonged neurogenesis and Ret upregulation providing a therapeutic target in ALK-mutated neuroblastoma
Source: Oncotarget. 2014 Apr 2;5(9):2688–702. doi: 10.18632/oncotarget.1883 (PMC4058037; doi:10.18632/oncotarget.1883)
Supplement: Supplementary file 1 [file oncotarget-05-2688-s001.pdf]

Supplementary Methods

Construction of the targeting vector for KI *Alk*^{R1279Q} mice

A fragment encompassing *Alk* exon 25 was amplified by polymerase chain reaction (PCR) to introduce the CGA > CAA point mutation (1275R > Q in human). As well, 5' and 3' homology arms were amplified by PCR. These fragments were cloned into a vector containing a neomycin resistance (Neo) cassette located between two LoxP sites.

Genotyping of KI *Alk*^{R1279Q} mice

PCR analysis of genomic tail biopsy DNA using primers Ef4639 (5' TAGATGCCTGGGCTGGCTTATATCTAA 3') and Er4640 (5' GAACTCGATTTCTGTGCCTCTGGG 3') was performed to discriminate Wt (357 bp) and KI (L-, 443 bp) alleles. The point mutation was confirmed by PCR using primers exon25-F (5' AACTGTTTGGACTTCACAGAAGG 3') and exon 25-R (5' TTGAGGAGACTGGCTGGTG 3') followed by Sanger sequencing.

Generation of KI *Alk*^{F1178L} mice

For the *Alk*^{F1178L} mutation, since a potential embryonal lethality was suspected, we initially planned to use a FLEx strategy to generate animals with a conditional expression of the mutated allele after Cre recombination (43). The FLEx system was expected to drive the expression of a mutated allele instead of the Wt allele after recombination of specific Lox sites flanking a mutated and inverted exon 23 placed in the intron following the normal exon 23. The targeting vector for *Alk*^{F1178L} was constructed as shown in Supplemental Figure 7 and contains the following parts: a 5' homology arm, the floxed arm (corresponding to exon 23 flanked by LoxP and Lox511 sites), the Flex arm (containing an inverted exon 23 exhibiting

the TTC > CTC point mutation (1174F > L in human) followed by a LoxP site), the FRT-Neo region (including the Neo cassette between two FRT sites and followed by a Lox511 site) and a 3' homology arm. Targeted 129Sv/Pas ES clones were confirmed by PCR and Southern blot and injected into C57BL/6J blastocysts to generate chimeric mice. Chimeras (L3) were crossed with transgenic flippase mice (C57BL/6) to check transmission of the targeted allele in the germline and excise the FRT site-flanked NeoR cassette on F1 progenies (L2). In order to confirm that this L2 allele gives a Wt Alk mRNA, we extracted RNA from brain of heterozygous mice and analyzed the transcript. We observed one band at the expected size (362 bp) as well as an additional band at a smaller size (~ 230 bp) (Supplemental Figure 6). Sanger sequencing of this band revealed that it corresponded to a mRNA in which the exon 23 (130 pb) was skipped. This lead to a frameshift resulting in the expression of a protein containing the amino acids 1 to 1175 of the mouse Alk receptor, followed by 25 additional amino acids before a STOP codon (Supplemental Figure 6). The L2 allele therefore drives the expression of a truncated Alk and not that of a Wt mRNA as expected. Consequently, we bred mice bearing the L2 allele with a transgenic CMV-Cre deleter line and obtained two males bearing the KI allele (L-). After segregation of the Cre transgene, we obtained one Alk^{F1178L} KI mice line.

PCR analysis of genomic tail biopsy DNA using primers Lf4735 (5' GTCGGCAGGAGATTTCAGAGACCA 3') and Mr4736 (5' GCAGGAGTTGAATTAGCGGGAAAAG 3') was performed to discriminate Wt (374 bp) and KI (L-, 449 bp) alleles. The point mutation was confirmed by PCR using primers exon23-F (5' CTTCACAGCGTGATTGCTGA 3') and exon 23-R (5' CAGTTACCTCCCTTGCAACC 3') followed by Sanger sequencing.

^{18}F[FDG] PET Imaging

PET acquisitions were performed with the Mosaic animal PET (Philips Medical systems, Cleveland, OH, USA). Mice were fasted for 12 h before ^{18}F[FDG] PET imaging but allowed free access to water. 5 MBq of the radiotracer were injected intravenously in the retro-orbital sinus of the animals. Mice were maintained under anesthesia with a mixture of 1.5% isoflurane and oxygen, and a heating lamp was used to keep body temperature during data acquisition. Static acquisitions were performed one hour after injection with an exposure time of 10 minutes. Images were reconstructed in 3-dimensional using PET view software (Philips Medical).

Reference

43. Schnutgen F, Doerflinger N, Calleja C, Wendling O, Chambon P, Ghyselinck NB. A directional strategy for monitoring Cre-mediated recombination at the cellular level in the mouse. Nature biotechnology. 2003;21:562–5.
