# Supplementary material for: Activated Alk triggers prolonged neurogenesis and Ret upregulation providing a therapeutic target in ALK-mutated neuroblastoma
Source: Oncotarget. 2014 Apr 2;5(9):2688–702. doi: 10.18632/oncotarget.1883 (PMC4058037; doi:10.18632/oncotarget.1883)
Supplement: Supplementary file 2 [file oncotarget-05-2688-s002.pdf]

Supplementary figure 1: Quantification of islet1-positive cells and double positive cells for islet1 and ki67 in SCG and stellate ganglia at E12.5.

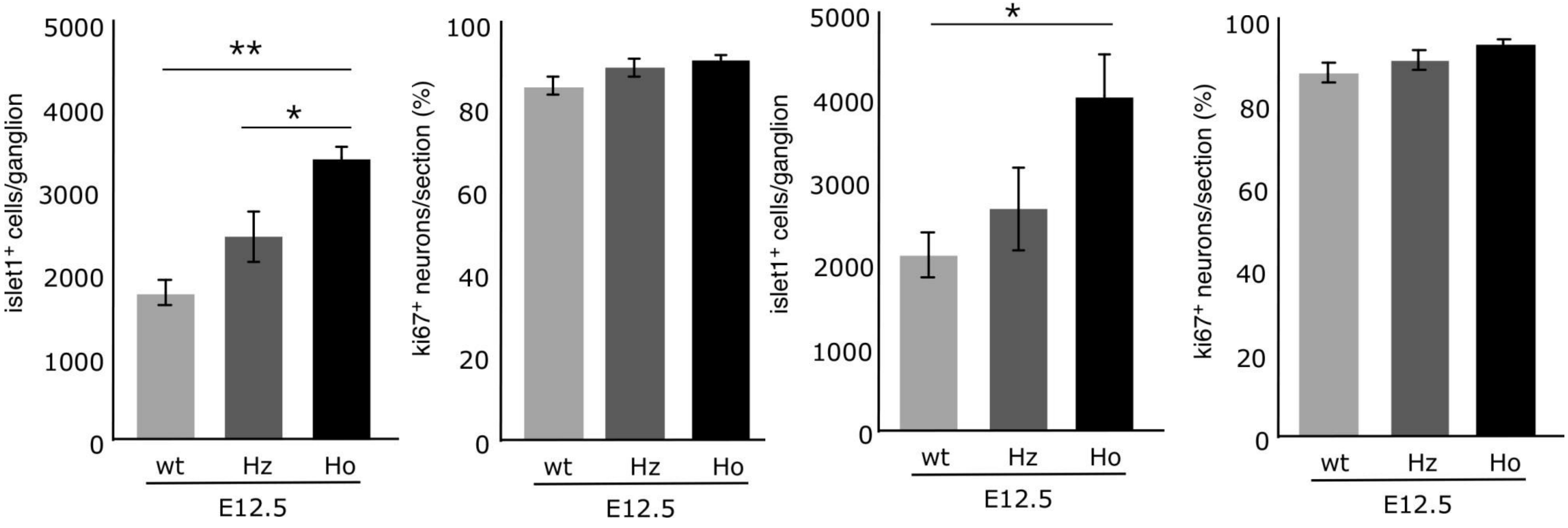

SCG

Stellate

Bonferroni Multiple Comparisons Tests were used to evaluate differences between the groups (n=5 samples in each group). Error bars represent standard deviation.

Supplementary figure 2: A. Boxplots showing expression of different genes in the 3 groups of PNS ganglia at birth. **B**. Boxplots showing expression of different genes in the 3 groups of tumors.

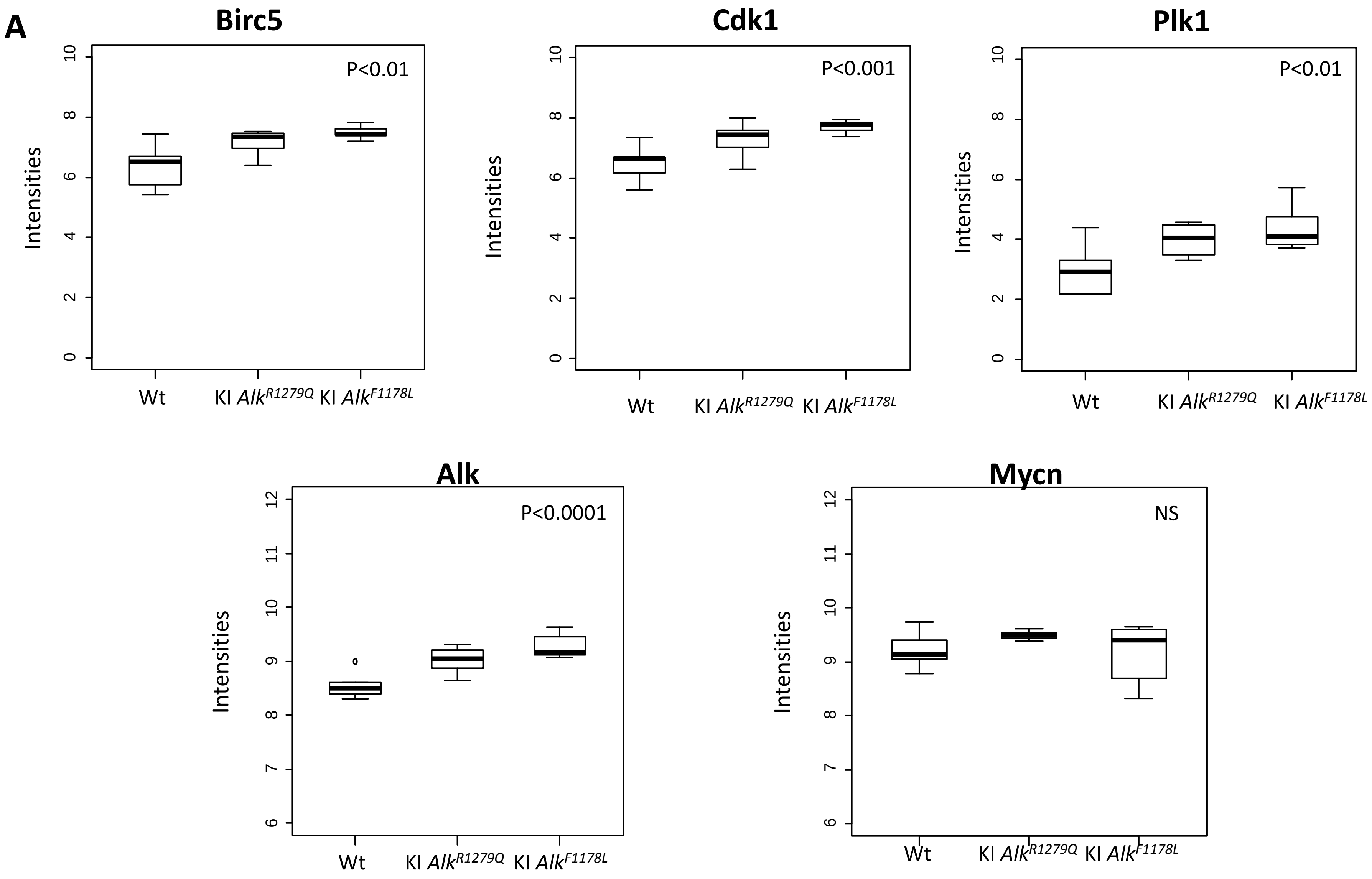

P-values were determined using a two-sided ANOVA test. (NS: not significant)

B

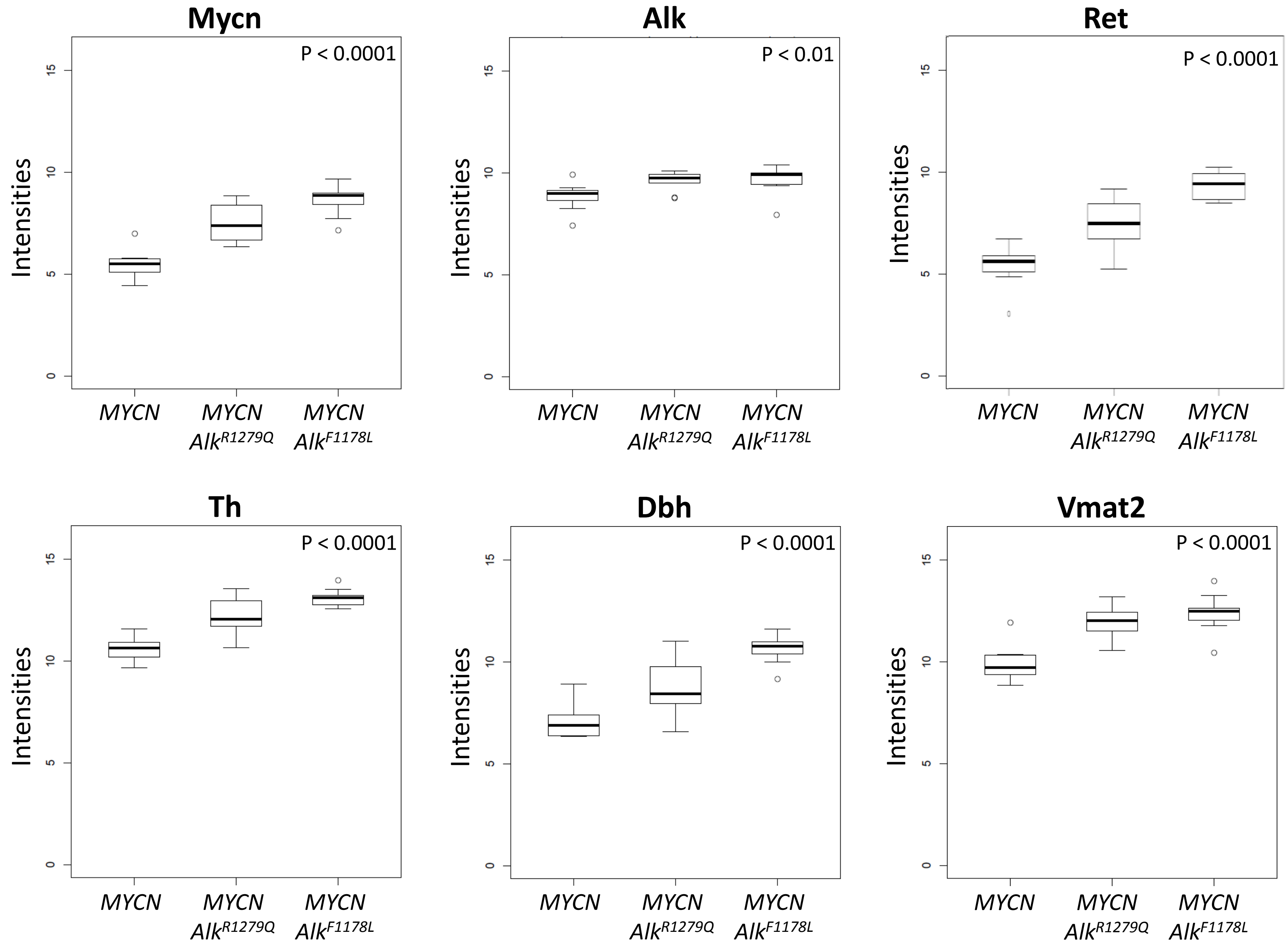

P-values were determined using a two-sided ANOVA test.

Supplementary figure 3: TH and ki67 stainings of *MYCN* and *MYCN/Alk* tumors. Representative figures are shown for each tumor group. (Left column: TH; right column: ki67)

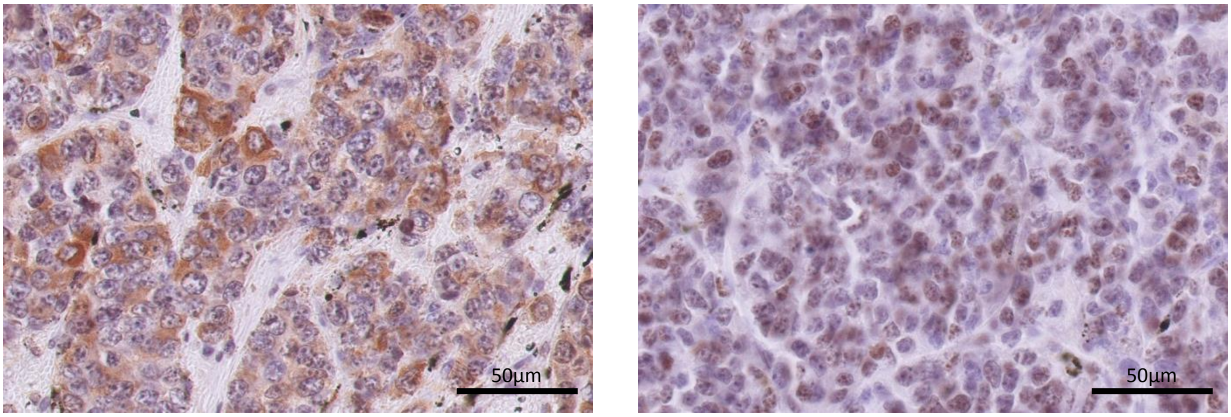

MYCN tumor

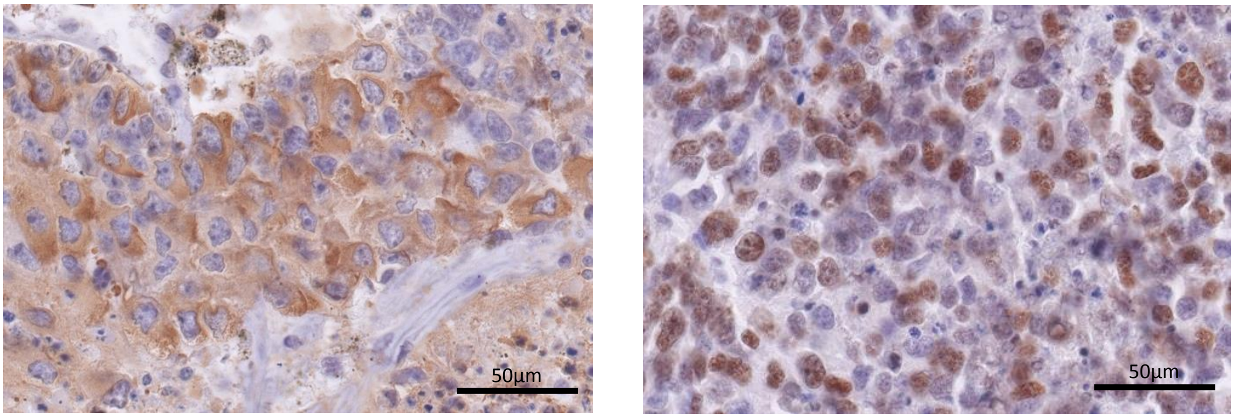

MYCN/Alk^{F1178L} tumor

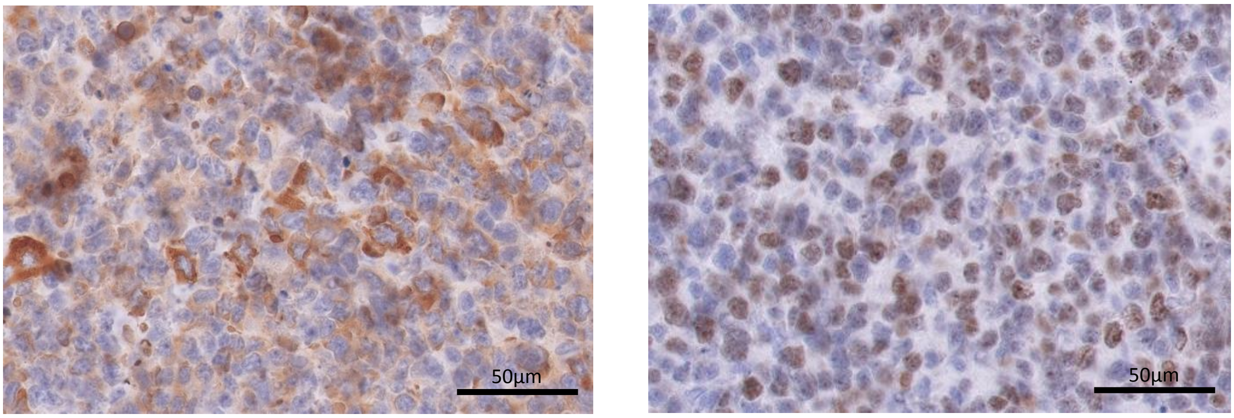

MYCN/Alk^{R1279Q} tumor

Supplementary Figure 4: Expression of Alk and MYCN proteins in tumors arising from *TH-MYCN*/KI *Alk* mice

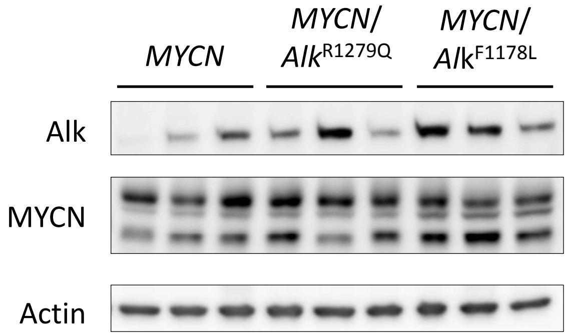

Two bands at 65 and 45 kD are detected for MYCN, consistently with the expected molecular weight of MYCN and ΔMYCN as previously described in the IMR32 neuroblastoma cell line (Jacobs et al, BMC Cancer, 2009).

MYCN and Alk were detected by immunoblotting using the #SC-791 (Santa Cruz) and #18-0266 (Life Technologies) antibodies, respectively.

Three tumors from different mice were analyzed for each genotype.

Supplementary figure 5: Expression of *VGF, VIP* and *DBH* upon ALK abrogation in human NB cell lines.

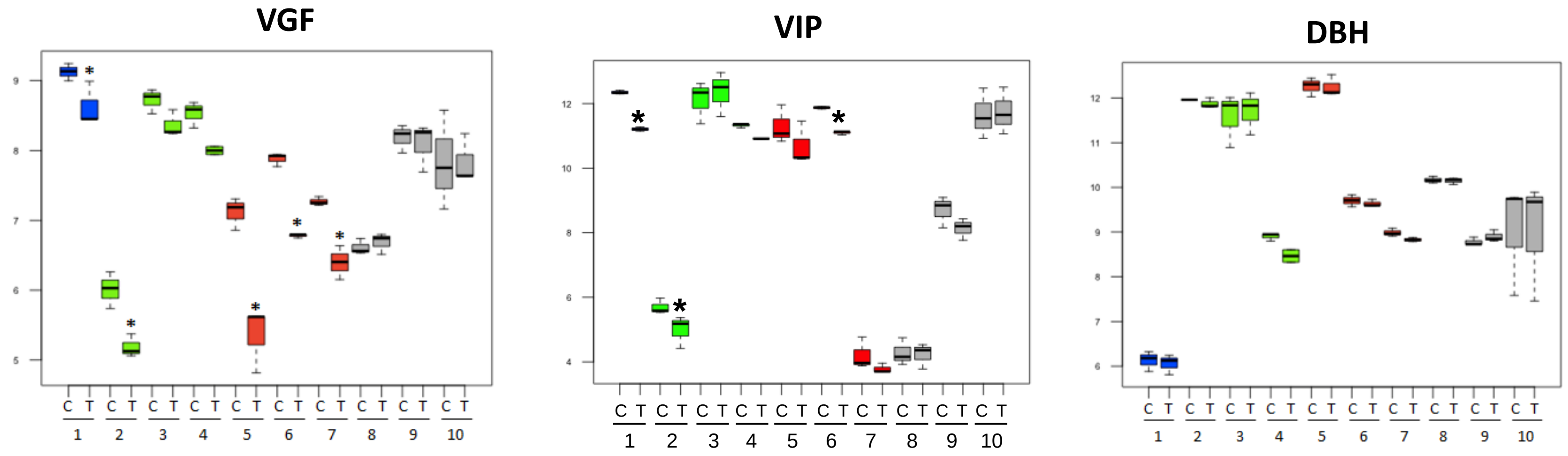

ALK amplification
ALK F1174L
ALK R1275Q
ALK Wt

1: NB-1
2: Kelly
3: SK-N-SH
4: SMS-KNCR
5: CLB-Ga
6: LAN5
7: UKF-NB-3
8: IMR-32
9: NGP
10: SK-N-AS

P values were calculated using one-sided Wilcox-test.

Supplementary Figure 6: Vegfr2 and Egfr proteins are not or hardly detected in tumors arising from *TH-MYCN*/KI *Alk* mice

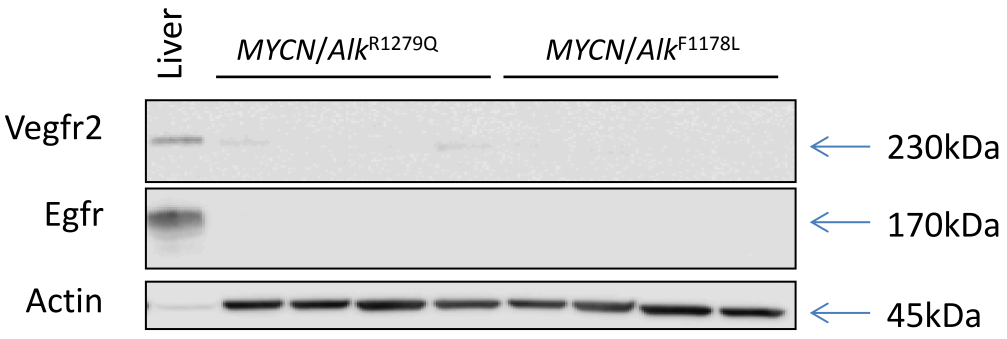

Vegfr2 and Egfr were detected by immunoblotting using the CST #2479 and #2232 antibodies, respectively.
Liver from a Wt mouse was used as a positive control.
Four tumors from different mice were analyzed for both genotypes.

Supplementary figure 7: Generation of KI *Alk*^{F1178L} mice. (a) Strategy used to generate the KI allele. (b) Skipping of exon 23 in the cDNA encoded by the conditional allele (L2). (c) Skipping of exon 23 results in the expression of a protein containing the amino acids 1 to 1175 of the mouse Alk receptor, followed by 25 additional amino acids before a STOP codon.

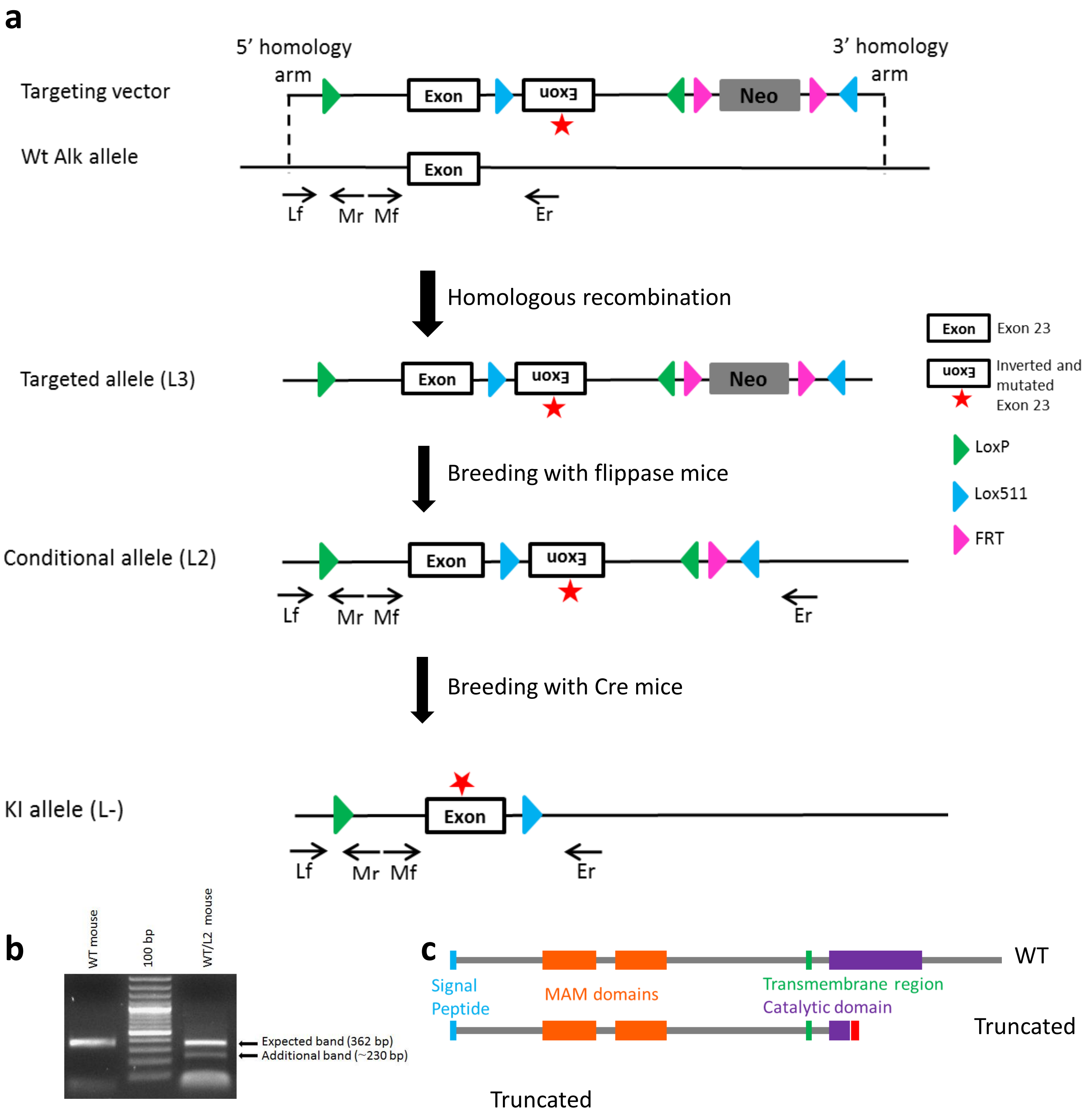

Truncated

```
1100 CFAGKTSSIS DLKEVPRKNI TLIRGLGHGA
1130 FGEVYEGQVS GMPNDPSPLQ VAVKTLPEVC
1160 SEQDELDFLM EALIIRTNPP PWPCWTFCMW
LGTLPVAVST
```
